# 

**Published:** 1897-07

**Authors:** 


					﻿Vol. XVII.
JULY, 1897.
NO. 7.
THE
pOMEOPATHlC pHY^lClAp,
A Monthly Journal of Homoeopathic Materia Medica
and Clinical Medicine.
“ He u a freeman whom truth makes free, and all are slaves betide."
"Seek the Truth: come whence it may, cost what it will."
EDITED AND PUBLISHED BY
WALTER Tvf. JAMES, M. E>.
PHILADELPHIA:
JSTo. 1231 LOCUST STREET.
LONDON:
ALFRED HEATH & CO., 8ole Agents for Great Britain,
114 Ebury Street, Eaton Square.
•Yearly Subscription, $2.SO, invariably in advance. Single numbers, 25 cts.
Entered at the Philadelphia Post-Office as Seeond-Class Mail Matter.
				

